# Impact of Menopause and the Menstrual Cycle on Oxidative Stress in Japanese Women

**DOI:** 10.3390/jcm12030829

**Published:** 2023-01-20

**Authors:** Ayaka Ishikawa, Hiroshi Matsushita, Saki Shimizu, Noriko Morita, Rina Hanai, Saeko Sugiyama, Kazushi Watanabe, Akihiko Wakatsuki

**Affiliations:** Department of Obstetrics and Gynecology, Aichi Medical University School of Medicine, Nagakute 480-1195, Aichi, Japan

**Keywords:** copper, estrogen, menopause, menstruation, oxidative stress, progesterone, reactive oxygen metabolites

## Abstract

Although estrogen possesses both pro- and anti-oxidant properties, its overall role in oxidative stress among women remains unclear, particularly since the influence of exogenously administered estrogen during previous studies differed by dose, administration route, and estrogen type. The aim of this study was to elucidate the effects of endogenous estrogen on oxidative stress in women. Thus, we performed a non-interventional observational study of healthy postmenopausal (*n* = 71) and premenopausal (*n* = 72) female volunteers. Serum levels of derivatives of reactive oxygen metabolites (d-ROMs, which are collectively a marker of oxidative stress), as well as the biological antioxidant potential (BAP, an indicator of antioxidant capacity), were compared between (1) pre- versus post-menopausal women, and (2) premenopausal women in early follicular versus mid-luteal phases of their menstrual cycles. We found that serum d-ROMs and BAP values in postmenopausal women were significantly higher than those in premenopausal women. Moreover, the d-ROM levels were significantly correlated with serum copper concentrations. However, neither d-ROMs nor BAP values were significantly affected by the menstrual cycle phase, although changes in d-ROMs between the follicular and luteal phases were significantly correlated with copper concentration shifts. These data indicate that postmenopausal hypoestrogenism is associated with elevated oxidative stress, although regular fluctuations of estrogen levels during the menstrual cycle do not influence oxidative stress.

## 1. Introduction

Oxidative stress is defined as an imbalance between the production of free radical reactive oxygen species (ROS) and a cell’s antioxidant response mechanisms [1]. It has been reported that elevated oxidative stress may be implicated in various diseases, such as atherosclerosis, chronic obstructive pulmonary disease, Alzheimer’s disease, and cancer [2]. To protect human biological systems from oxidative stress, the production of ROS is regulated by several antioxidant defense mechanisms that include both enzymatic and non-enzymatic pathways [3,4]. Antioxidant enzyme activity is influenced by various factors, such as age, organ specificity, and hormonal status [5]. Because hormones regulate metabolic activities requiring oxygen in aerobic cells and considering that the incomplete reduction of oxygen produces ROS, alterations in hormonal status may have an impact on the latter’s production [5]. Additionally, certain hormones can themselves act as antioxidants and can have an impact on various enzymatic and non-enzymatic components of the antioxidant response system [5].

Estrogens, including estrone (E1), estriol (E3), and biologically active metabolite 17ß-estradiol (E2), are sex steroid hormones that are primarily produced in the ovaries and play an important role in regulating female reproductive functions. Menopause is associated with an increase in oxidative stress [6,7,8], suggesting that estrogens may have antioxidant properties. In previous studies, E1, E2, and E3 inhibited microsomal lipid peroxidation in vitro [9], and all estrogens had an inhibitory effect on low-density lipoprotein (LDL) oxidation in vitro (with E2 being the most inhibitory followed by E1, equilin, and E3) [10]. In human studies, Sack et al. [11] found that the physiological levels of E2 had an inhibitory effect on LDL oxidation in postmenopausal women. Additionally, Wakatsuki et al. [12] reported that conjugated equine estrogen inhibited the susceptibility of LDL and high-density lipoprotein to oxidative modification in postmenopausal women. In contrast, some studies found that exogenously administered estrogen exerted pro-oxidant properties. For example, Pincemail et al. [13] reported that the intake of oral contraceptives significantly increased lipid peroxidation in women aged 40–48 years. De Groote et al. [14] also demonstrated that oral contraceptives containing 0.03 mg of ethinylestradiol and 3 mg of drospirenone significantly increased the mean levels of lipid peroxides and oxidized LDLs.

The age-related reduction in antioxidant activity accompanied by increased oxidative stress increases oxidative LDL [15]. Furthermore, oxidative stress is implicated in the pathogenesis of various age-related diseases. Menopause is associated with an increase in oxidative stress, which makes women more susceptible to various chronic diseases, such as atherosclerosis, Alzheimer’s disease, and postmenopausal osteoporosis [16]. Recently, we evaluated oxidative stress by analyzing derivatives of reactive oxygen metabolites (d-ROMs) and the biological antioxidant potential (BAP) in postmenopausal women treated with three different types of estrogen therapy. We found that d-ROMs were significantly increased by the oral intake of conjugated equine estrogen, and this increase was significantly greater than the transdermal or oral E2 [17]. LDL oxidation was not inhibited by the oral intake of conjugated equine estrogen. In contrast, d-ROMs remained unchanged by an oral E2 and were decreased by the transdermal E2 [17]. These findings suggest that the effect of exogenously administered estrogen on oxidative stress and LDL oxidation may differ according to the administration route and/or estrogen type [17].

This study aimed to elucidate the effects of endogenous estrogen on oxidative stress in women. We compared d-ROM levels and BAP values according to the physiological estrogen status, namely, between pre- and post-menopausal women as well as between premenopausal women in the follicular versus luteal phases of their menstrual cycles.

## 2. Materials and Methods

### 2.1. Participants

A total of 71 healthy postmenopausal women and 72 healthy eumenorrheic premenopausal women with 25-to-38-day cycles volunteered in this non-interventional observational study conducted at the Department of Obstetrics and Gynecology of Aichi Medical University Hospital, Nagakute, Japan. To minimize the effect of the aging factor on the results, postmenopausal women aged 45–60 years within 5 years after menopause and premenopausal women aged 20–40 years were included.

The sample size calculation was determined a priori using the G*Power software 3.1.9 for Windows (Heinrich Heine-Universität Düsseldorf, Düsseldorf, Germany). With the power calculated at 0.8, an α set at a two-sided level of 0.05, and the effect size d set at 0.5, a sample size of 67 per group was found to be necessary for the Mann–Whitney U-test to detect differences between premenopausal and postmenopausal women, whereas a sample size of 35 was sufficient for the Wilcoxon rank-signed test to detect differences between early follicular and luteal phases in premenopausal women during menstruation. The study was approved by the Institutional Review Board of Aichi Medical University (approval no. 11-079) and was conducted in accordance with the Declaration of Helsinki. All participants provided written informed consent before enrollment in the study.

Data on current ages, ages at menarche or menopause, and menstrual cycles were collected at enrollment. The exclusion criteria were women with chronic or metabolic diseases; those who had undergone surgical menopause; and/or those who had taken medications that could influence the study’s outcomes, such as hormone replacement therapy, oral contraceptives, statins, fibrates, and/or multivitamin/mineral supplements. Women who engaged in excessive caffeine or alcohol intake, as well as those who were smokers, were also excluded. Premenopausal women were asked to report the first day of any subsequent menstruation.

### 2.2. Measurements

Height and weight were measured with a portable stadiometer and body scale, respectively. The body mass index (BMI) was calculated as the weight in kilograms divided by the square of the height in meters (kg/m^2^).

Early morning venous blood was drawn from postmenopausal women upon enrollment after overnight fasting and during days 2–5 of the early follicular phase and day 21 ± 2 of the luteal phase of the menstrual cycle. The participants were instructed to avoid intense exercise, caffeine, and high-antioxidant-containing foods or beverages for 24 h before the blood drowning. Immediately after removing the sera, the d-ROMs (a marker of oxidative stress) and BAP values (an indicator of antioxidant protein levels) were determined using the Free Radical Analytical System version 4 (H&D S.r.l., Parma, Italy) as previously described [17,18]. This assay measures the serum levels of organic hydroperoxides, reflecting the levels of free radicals from which they were formed. Twenty microliters of serum sample was dissolved in an acidic buffer, where hydroperoxides were converted to alkoxyl and peroxyl radicals by catalytic oxidation with iron ions liberated from proteins. Then, the samples were transferred to new tubes, where N,N-diethyl-paraphenylenediamine, one of the chromogens, was oxidized by alkoxyl and peroxyl radicals. The oxidized N,N-diethyl-paraphenylenediamine was colored a reddish violet. Absorption was determined spectrophotometrically at 505 nm, which corresponded to the number of d-ROMs. The BAP test measures the serum’s antioxidant potential, which is indicative of antioxidant levels. Ten microliters of serum sample was mixed with a colored solution containing ferric ions bound to a chromogenic substrate (thiocyanate derivative) and incubated at 37 °C for 5 min. When ferric ions were reduced to ferrous ions by anti-oxidant substrates, the thiocyanate derivative was decolored. Absorption at 505 nm was also measured, which corresponded to the amount of BAP. Measurements obtained from the d-ROMs and BAP tests were expressed as Carratelli units (CARR U), with 1 CARR U corresponding to 0.08 mg/100 mL H_2_O_2_ and μmol/L, respectively. The oxidative stress index (OSI) was calculated as OSI = d-ROMs/BAP × 100 (CURR U/μmol/L).

The remaining sera were aliquoted and stored at −80 °C; to avoid inter-assay variance, all samples were analyzed in batches at the Hachioji Laboratory of SRL, Inc. (Tokyo, Japan). Estradiol levels were measured using a chemiluminescent immunoassay (intra-assay coefficient of variability [CV], 1.7–3.7%; inter-assay CV, 1.2–3.1%). Progesterone levels were measured using an electrochemiluminescence immunoassay (intra-assay CV, 2.0–4.2%; inter-assay CV, 1.7–3.5%). Copper levels were measured using a colorimetric method (intra-assay CV, 0.9–2.2%; inter-assay CV, 0.4–0.9%). Iron levels were measured using the 2-nitroso-5-(N-n-propyl-N-[3-sulfopropyl] amino) phenol method (intra-assay CV, 1.4–1.9%; inter-assay CV, 0.0–0.0%). LDL was measured using an enzyme-linked immune-sorbent assay (intra-assay CV, 4.7–5.7%; inter-assay CV, 5.2–5.6%).

### 2.3. Statistical Analysis

The data are expressed as the mean ± standard deviation. All data management and statistical analyses were performed using JMP version 12.2.0 (SAS Institute Inc., Cary, NC, USA). A *p*-value < 0.05 was considered statistically significant.

For cross-sectional analyses, comparisons between postmenopausal and premenopausal women (days 2–5) were performed using the Mann–Whitney U-test. Multiple regression analyses were performed to evaluate the contribution of age, height, weight, serum copper, iron, and oxidized LDL on the d-ROMs. If available, the years since menopause and the serum estradiol and progesterone levels were also included as variables.

For longitudinal analyses, the differences between early follicular and luteal phases in premenopausal women were analyzed using the Wilcoxon rank-signed test. To identify independent predictors of d-ROM shifts (Δd-ROMs), multiple regression was performed to evaluate the contribution of the changes in BAP (ΔBAP), estradiol (ΔEstradiol), progesterone (ΔProgesterone), copper (ΔCopper), iron (ΔIron), and oxidized LDL (ΔOxidized LDL). The d-ROMs value (day 2) was included as an independent variable to adjust for regression toward the mean.

## 3. Results

Four premenopausal women were excluded from the final analysis because they reported smoking during the study period (*n* = 2) or experienced an unexpected delay in their upcoming menstruation (*n* = 2). Ultimately, 71 postmenopausal and 68 premenopausal women were included, and their characteristics are presented in Table 1. The mean ages of the postmenopausal and premenopausal women were 53.7 (median, 54; range, 48–60) and 25.3 (median, 24; range, 21–37) years, respectively. The weight, BMI, and blood pressure of postmenopausal women were significantly higher than those of premenopausal women. In addition, serum d-ROMs and BAP levels, and OSI among postmenopausal women were significantly higher than among premenopausal women. The serum iron, copper, and oxidized LDL levels were also significantly higher among postmenopausal women.

Among premenopausal women, serum concentrations of estradiol and progesterone were significantly higher during the luteal phase than during the follicular phase. There were no significant differences in the serum d-ROMs and BAP levels between the early follicular and luteal phases. The serum copper and oxidized LDL levels were significantly lower in the luteal phase, while serum iron levels were significantly higher.

We performed multiple regression analyses to examine the contributions of age; years since menopause; height; weight; BAP; and serum estradiol, progesterone, iron, copper, and oxidized LDL levels on serum d-ROMs (Table 2). We found that the d-ROMs level was positively correlated with serum copper concentration in postmenopausal women. In premenopausal women, the d-ROMs was positively correlated with copper in the follicular phase and with both copper and BAP in the luteal phase. However, neither estradiol nor progesterone was correlated with the concentration of d-ROMs in the follicular or luteal phase.

Multiple regression analyses were also performed to determine the contribution of ΔBAP, ΔEstradiol, ΔProgesterone, ΔCopper, ΔIron, and ΔOxidized LDL on the Δd-ROMs during the menstrual cycle. These demonstrated that the ΔBAP and ΔCopper levels were positively correlated with the Δd-ROMs. However, neither the ΔEstradiol nor ΔProgesterone levels were correlated with the Δd-ROMs during the menstrual cycle (Table 3).

## 4. Discussion

Although it has been demonstrated that estrogen possesses pro- and anti-oxidant properties, its role in oxidative stress among women remains debated. Thus, we explored the influence of endogenous estrogen on oxidative stress by comparing markers of the latter as a function of physiological estrogen fluctuations.

First, we demonstrated that the serum levels of d-ROMs (a marker of oxidative stress) and BAP (an indicator of antioxidant protein levels) were significantly higher in postmenopausal women than in premenopausal women. Previous studies investigating the influence of natural menopause on oxidative status have produced inconsistent findings [6,7,19,20,21,22,23,24]. Our data are consistent with the findings of Montoya-Estrada et al. [24], who showed that postmenopausal Mexican women exhibited significantly higher oxidative stress markers (including diene conjugates, lipohydroperoxides, malondialdehyde, and carbonylated proteins) compared with reproductive-aged women and that they also had a higher total antioxidant capacity (TAC). Signorelli et al. [19] also revealed that malondialdehyde, 4-hydroxynenal, and oxidized LDL were significantly higher in postmenopausal women than in fertile women, although glutathione peroxidase levels were significantly lower. In contrast, Pansini et al. [21] demonstrated that d-ROM levels were not significantly influenced by menopausal status, although antioxidant status, as determined by the ferric-reducing antioxidant power method, was significantly higher in postmenopausal women. Furthermore, Brunelli et al. [25] found no significant differences in either serum d-ROMs or BAP levels between pre- and post-menopausal women.

The reasons for the discrepancies between these studies remain unclear. In our current investigation, there were significant differences in age, body weight, blood pressure, and serum iron, copper, and oxidized LDL levels; these inevitable heterogeneities associated with menopause may have influenced the results [6,21,26,27]. However, our multiple regression analysis revealed that only serum copper concentrations were significantly correlated with d-ROM levels, whereas age, years since menopause, height, weight, serum BAP, serum iron, and oxidized LDL levels did not. Signorelli et al. [19] also found no correlation between oxidative stress markers (such as malondialdehyde, 4-hydroxynenal, oxidized LDL, and glutathione peroxidase) and total cholesterol, LDL, BMI, or age per univariate and multivariate analyses. A longitudinal study comparing premenopausal women before versus after undergoing bilateral oophorectomy, or a cross-sectional analysis comparing premenopausal women with age-matched surgically menopausal counterparts, may help avoid potential biases owing to these factors (although such studies are extremely rare). Bellanti et al. [28] reported that surgical menopause was associated with an increase in oxidized glutathione levels and a decrease in reduced blood glutathione levels with lower superoxide dismutase and glutathione peroxidase mRNA expression, suggesting that menopause (a hypoestrogenic state) may lead to increased oxidative stress.

Second, our current study revealed no significant differences in serum d-ROM levels and BAP values between women in the early follicular versus mid-luteal phases of their menstrual cycles, suggesting that estrogen fluctuations during the cycle may not impact oxidative status. Although there are few studies investigating the influence of the menstrual cycle on oxidative stress, several investigators have reported conflicting findings. Iida et al. [29] and Konishi et al. [30] reported that the concentration of urinary 8-hydroxy-2′ deoxyguanosine in young female university students was not influenced by the menstrual cycle. In contrast, Karowicz-Bilinska et al. [31] reported higher levels of urinary hydrogen peroxide and thiobarbituric acid reactive substances in the luteal phase of the cycle. Furthermore, Cornelli et al. [32] assessed the serum d-ROM values every 3 days during the menstrual cycle and found that they were significantly elevated from day 6 to 24 compared to day 1. However, they found no correlations between d-ROMs and E2, and speculated that the increase in systemic oxidation is not determined exclusively by E2 but also involves other factors.

Premenopausal women produce progesterone in a cyclical manner, and it has also been reported that high levels of progesterone may reduce oxidative damage [5]. However, our multiple regression analysis revealed that neither E2 nor progesterone was correlated with the serum d-ROM levels in the follicular and luteal phases. Additionally, neither the fluctuation in estradiol (ΔEstradiol) nor progesterone (ΔProgesterone) was associated with the fluctuation in d-ROMs (Δd-ROMs) during the menstrual cycle. Santanam et al. [33] investigated the inhibition of the copper-mediated oxidation of LDL as a function of serum E2 levels by measuring the latter serially in women before ovulation (days 1, 7, 9, and 12) and found no significant inhibition of LDL oxidation even at peak E2 concentrations. They also found that the elevated E2 following ovarian hyperstimulation for in vitro fertilization inhibited oxidation, and they concluded that the physiologic levels of E2 may not be sufficient to exhibit antioxidant properties. Taken together, these data indicate that endogenous estrogen has little influence, if any, on d-ROMs in premenopausal women. However, the results presented herein should be interpreted with caution since we did not determine the follicular and luteal phases of the menstrual cycle using more accurate methods, such as ultrasonography. In addition, premenopausal women in the present study were young (mean age of 25.32 ± 3.63 years); therefore, the influences associated with age-related increases in oxidative stress and oxidized LDL may have been eliminated.

Our study also revealed that only serum copper concentrations were significantly correlated with d-ROMs in postmenopausal and (both follicular- and luteal-phase) premenopausal women. Additionally, although there was a significant change in serum iron concentration, the Δd-ROMs during the menstrual cycle were significantly correlated with the ΔCopper levels. Michos et al. [34] identified a cyclical fluctuation in plasma copper concentrations during the menstrual cycle in healthy eumenorrheic women that was negatively correlated with the E2 concentration. Additionally, Mazzetti et al. [35] demonstrated that plasma lipid peroxide levels were positively correlated with copper concentrations. d-ROM levels reflect oxidative stress because they indicate reactive oxygen metabolite levels, mainly hydroperoxides (hydroxyl radicals) that are oxidized by ROS. Hydroxyl radicals are formed in biological systems when transition-metal ions, such as copper or iron ions, participate in a Fenton reaction [17]. Taken together, we speculate that the influence of endogenous estrogen on oxidative stress might be indirectly reflected by copper levels.

Notably, the present study has some limitations. First, in the present study, to minimize the effect of age, postmenopausal women were limited to 45–60 years within 5 years after menopause and premenopausal women to 20–40 years. However, the influences affected by age could not be fully excluded. Although multiple regression analyses failed to show a significant correlation between age and d-ROMs in post- and pre-menopausal women, women of the same age should have been included in post- and pre-menopausal groups. Second, in the present study, we evaluated only copper and iron levels as trace elements. Previously, we evaluated d-ROMs and BAP levels in postmenopausal women treated with three types of estrogen and found that the change in d-ROMs was significantly influenced by the copper concentration, although the change was not influenced by the zinc concentration at all [17]. However, previous studies reported that other trace elements, such as bromine, cobalt, iodine, molybdenum, nickel, and vanadium, may affect d-ROM concentrations [36]. Third, because we could not assess the influences of diet, the findings of the present study might be influenced by the dietary copper content consumed by the participants. Additional research is needed to investigate the effect of dietary copper on oxidative stress.

## 5. Conclusions

We found that oxidative stress, as evaluated by d-ROMs, was significantly higher in postmenopausal women than in premenopausal counterparts. However, there were no significant shifts in oxidative stress markers during the menstrual cycle. Additional studies are needed to fully understand the mechanisms by which endogenous estrogen may regulate oxidative stress in women.

## Figures and Tables

**Table 1:** Characteristics and biochemical parameters of the postmenopausal and premenopausal women.

Variables	Postmenopausal (*n* = 71)	Premenopausal (*n* = 68)	
		Follicular Phase	Luteal Phase
Age (years)	53.73 ± 2.68 ^a^	25.32 ± 3.63	
Age at menarche (years)	12.63 ± 1.44 ^b^	12.03 ± 1.46	
Age at menopause (years)	51.14 ± 2.42		
Years since menopause	2.59 ± 1.72		
Menstrual cycle (day)		29.18 ± 2.11	
Height (cm)	158.20 ± 5.85	158.40 ± 4.89	
Weight (kg)	54.92 ± 8.45 ^a^	49.05 ± 4.86	
Body mass index (kg/m^2^)	22.01 ± 3.32 ^a^	19.54 ± 1.57	
Systolic BP (mmHg)	122.44 ± 19.50 ^a^	110.72 ± 9.54	
Diastolic BP (mmHg)	72.72 ± 13.24 ^b^	67.69 ± 9.18	
Estradiol (pg/mL)	N/A	37.60 ± 21.98	165.90 ± 98.12 ^c^
Progesterone (ng/mL)	N/A	0.169 ± 0.105	8.836 ± 7.764 ^c^
d-ROMs (CARR U)	385.27 ± 76.76 ^a^	260.25 ± 64.32	251.19 ± 48.80
BAP (μmol/L)	2655.21 ± 359.80 ^b^	2529.17 ± 265.39	2501.40 ± 300.44
OSI (CURR U/μmol/L)	14.62 ± 2.77 ^a^	10.38 ± 2.55 ^b^	10.08 ± 1.68
Iron (μg/dL)	103.30 ± 29.89 ^a^	69.78 ± 34.49	82.96 ± 40.10 ^d^
Copper (μg/dL)	105.63 ± 14.70 ^a^	93.87 ± 16.54	92.04 ± 13.49 ^d^
Oxidized LDL (U/L)	142.93 ± 47.79 ^a^	85.18 ± 20.77	79.31 ± 21.92 ^d^

Data are expressed as mean ± standard deviation of the mean. BAP, biological antioxidant potential; BP, blood pressure; d-ROMs, derivatives of reactive oxygen metabolites; N/A, not available; and OSI, oxidative stress index. ^a^ *p* < 0.01 compared with premenopausal follicular phase (Mann–Whitney U-test); ^b^ *p* < 0.05 compared with premenopausal follicular phase (Mann–Whitney U-test); ^c^ *p* < 0.01 compared with premenopausal follicular phase (Wilcoxon rank-signed test); and ^d^ *p* < 0.05 compared with premenopausal follicular phase (Wilcoxon rank-signed test).

**Table 2:** Multiple regression models examining the influences of several variables on d-ROMs in postmenopausal and premenopausal women.

Variables	Postmenopausal (*n* = 71)				Premenopausal (*n* = 68)							
					Follicular Phase				Luteal Phase			
	Estimates	SEM	*t* Ratio	*p*-Value	Estimates	SEM	*t* Ratio	*p*-Value	Estimates	SEM	*t* Ratio	*p*-Value
Constant	−76.45	272.64	−0.28	0.78	−391.12	213.63	−1.83	0.07	−94.41	161.37	−0.59	0.56
Age (years)	−3.84	3.30	−1.16	0.25	−1	1.47	−0.68	0.5	0.48	1.25	0.38	0.7
Years since menopause	3.35	5.05	0.66	0.51	N/A	N/A	N/A	N/A	N/A	N/A	N/A	N/A
Height (cm)	1.39	1.33	1.05	0.30	2.22	1.40	1.58	0.12	0.12	1.11	0.10	0.92
Weight (kg)	0.20	1.02	0.19	0.85	−0.88	1.35	−0.65	0.52	−0.33	1.08	−0.30	0.76
BAP (μmol/L)	0.07	0.02	3.16	<0.01	0.03	0.02	1.30	0.2	0.05	0.02	3.13	<0.01
Estradiol (pg/mL)	−	−	−	−	−0.06	0.25	−0.22	0.82	0.04	0.04	0.87	0.39
Progesterone (ng/mL)	−	−	−	−	70.29	51.62	1.36	0.18	0.63	0.65	0.97	0.33
Iron (μg/dL)	−0.34	0.25	−1.38	0.17	0.08	0.16	0.51	0.61	0.16	0.11	1.38	0.17
Copper (μg/dL)	2.84	0.57	4.95	<0.01	3.02	0.33	9.10	<0.01	2.26	0.34	6.58	<0.01
Oxidized LDL (U/L)	−0.08	0.16	−0.50	0.62	0.04	0.28	0.14	0.89	−0.25	0.21	−1.20	0.24
	Adjusted R^2^ = 0.41; *p* < 0.01				Adjusted R^2^ = 0.60; *p* < 0.01				Adjusted R^2^ = 0.55; *p* < 0.01			

Dependent variable = d-ROMs. BAP, biological antioxidant potential; d-ROMs, derivatives of reactive oxygen metabolites; LDL, low-density lipoprotein; N/A, not applicable; and SEM, standard error of the mean.

**Table 3:** Multiple regression models examining the influences of changes in various measures on shifts in d-ROMs in premenopausal women.

Variables	Estimates	SEM	*t* Ratio	*p*-Value
Constant	147.37	26.94	5.47	<0.01
d-ROMs (D2–5) (CARR U)	−0.62	0.09	−6.75	<0.01
ΔBAP (μmol/L)	0.04	0.01	2.90	<0.01
ΔEstradiol (pg/mL)	−0.01	0.05	−0.27	0.79
ΔProgesterone (ng/mL)	0.63	0.67	0.94	0.35
ΔIron (μg/dL)	0.19	0.14	1.36	0.18
ΔCopper (μg/dL)	1.35	0.54	2.25	<0.05
ΔOxidized LDL (U/L)	−0.37	0.26	−1.41	0.16
	Adjusted R^2^ = 0.60; *p* < 0.01			

Dependent variable = Δd-ROMs. BAP, biological antioxidant potential; CARR U, Carratelli units; D2–5, days 2–5; d-ROMs, derivatives of reactive oxygen metabolites; LDL, low-density lipoprotein; and SEM, standard error of the mean.

## Data Availability

The datasets generated during and/or analyzed during the current study are available from the corresponding author upon reasonable request.
